# A novel cryptic splice site mutation in *COL1A2* as a cause of osteogenesis imperfecta

**DOI:** 10.1016/j.bonr.2021.101110

**Published:** 2021-07-26

**Authors:** Ahmed El-Gazzar, Johannes A. Mayr, Barbara Voraberger, Karin Brugger, Stéphane Blouin, Katharina Tischlinger, Hans-Christoph Duba, Holger Prokisch, Nadja Fratzl-Zelman, Wolfgang Högler

**Affiliations:** a Department of Paediatrics and Adolescent Medicine, Johannes Kepler University Linz, Linz, Austria; b Department of Paediatrics, University Hospital Salzburg, Paracelsus Medical University Salzburg, Salzburg, Austria; c Ludwig Boltzmann Institute of Osteology at Hanusch Hospital of OEGK and AUVA Trauma Centre Meidling, 1^st^ Medical Department Hanusch Hospital, Vienna, Austria; d Institute of Medical Genetics, Med Campus IV, Kepler University Hospital, Linz, Austria; e Institute of Human Genetics, Technische Universität München, Munich, Germany; f Helmholtz Zentrum München, Institute of Neurogenomics, Munich, Germany

**Keywords:** Bone fragility, Type I collagen, Osteogenesis imperfecta, Cryptic splice site, Mutation, Whole exome sequencing

## Abstract

Osteogenesis imperfecta (OI) is an inherited genetic disorder characterized by frequent bone fractures and reduced bone mass. Most cases of OI are caused by dominantly inherited heterozygous mutations in one of the two genes encoding type I collagen, *COL1A1* and *COL1A2*. Here we describe a five-year-old boy with typical clinical, radiological and bone ultrastructural features of OI type I. Establishing the molecular genetic cause of his condition proved difficult since clinical exome and whole exome analysis was repeatedly reported negative. Finally, manual analysis of exome data revealed a silent *COL1A2* variant c.3597 T > A (NM_000089.4), which we demonstrate activates a cryptic splice site. The newly generated splice acceptor in exon 50 is much more accessible than the wild-type splice-site between the junction of exon 49 and 50, and results in an in-frame deletion of 24 amino acids of the C-terminal propeptide. In vitro collagen expression studies confirmed cellular accumulation and decreased COL1A2 secretion to 45%. This is the first report of a cryptic splice site within the coding region of *COL1A2.* which results in abnormal splicing causing OI. The experience from this case demonstrates that routine diagnostic approaches may miss cryptic splicing mutations in causative genes due to the lack of universally applicable algorithms for splice-site prediction. In exome-negative cases, in-depth analysis of common causative genes should be conducted and trio-exome analysis is recommended.

## Highlights

• Our study highlights a new category of mutations found in *COL1A2*.
• Manual screening of WES data identified the cryptic splice site in *COL1A2*
• NGS may miss cryptic splicing mutations in causative genes
• Universally applicable algorithms for splice-site prediction are needed

## 1 Introduction

Osteogenesis imperfecta (OI, MIM: PS166200), an inherited disorder of connective tissue, is a genetically heterogeneous skeletal dysplasia characterized by low bone mass, increased bone fragility, recurrent fractures, skeletal deformities and extraskeletal manifestation often accompanied by short stature (Forlino and Marini, 2016; Marini et al., 2017).

Approximately 85–90% of OI patients have dominant pathogenic variants in *COL1A1* (MIM: 120150) and *COL1A2* (MIM: 120160), which encode the alpha 1 and alpha 2 chains of type I collagen, respectively. To date, mutations in 24 genes have been identified as cause of OI (El-Gazzar and Hogler, 2021). Their encoded gene products exhibit functions in the nucleus, endoplasmic reticulum, Golgi apparatus, cytoskeleton, or in the extracellular matrix. At present, all genotypes manifest as five clinical phenotypes of OI (Forlino and Marini, 2016).

There are many pathogenic variants that have been identified in type I collagen genes, e.g. in *COL1A2,* by May 2021, 730 mutations were delineated (https://databases.lovd.nl/shared/genes/COL1A2). Among these, 83 variants were reported as alterations in splice-sites representing the second most common type of mutation (Kuivaniemi et al., 1997). Splice site mutations may result in exon skipping, intronic inclusion, or activation of cryptic sites in introns or exons (Marini et al., 2007). Very occasionally, cryptic splice sites are activated as a result of a nucleotide change by mutation. These dormant sites are often adjacent to authentic splice sites (Green, 1986; Padgett et al., 1986). Once activated, these sites become very efficiently expressed causing genetic diseases (Buratti et al., 2011; Buratti et al., 2007; Wang and Cooper, 2007). The consequence for the mRNA transcription and protein translation depend on whether these alterations are in-frame or produce a translational frameshift. To date, no cryptic splice site mutations have been described in *COL1A2*.

A variety of techniques are used to identify variants in genes typically associated with bone fragility. Following the clinical diagnosis of OI, the first approach usually involves OI gene panels. If the gene panel does not reveal a pathogenic variant, an exome wide analysis using whole exome sequencing (WES) or even whole genome sequencing (WGS), should be considered. If no pathogenic variant that can reasonably explain the patient's bone phenotype can be identified by WES analysis, this might be due to limitations in diagnostic algorithms. Especially for recognizing splice-relevant variants due to a lack of universally applicable algorithms for splice-site prediction (Park et al., 2018). Remarkably, splice-site mutations can occur in introns and exons.

Here, we describe a de novo cryptic splice site mutation in *COL1A2* which results in a new splice acceptor site leading to deletion of 72 nucleotides (24 amino acids). This pathogenic, in-frame deletion, variant escaped detection by two WES attempts since the alteration in the nucleotide did not change the amino acids, due to creation of a new splice acceptor site within the coding region.

## 2 Material and methods

### 2.1 Clinical data, bone histomorphometry and quantitative backscattered electron microscopy

All clinical data were obtained from the medical records and bone, skin and all blood samples were obtained with written informed consent of the patient's parents. A transiliac bone biopsy sample was taken following tetracycline labelling and analyzed by histomorphometry and quantitative backscattered electron microscopy (qBEI) using standard procedures previously described (Fratzl-Zelman et al., 2009; Glorieux et al., 2000; Mahr et al., 2021)*.* qBEI was performed with a field emission scanning electron microscope (SEM SUPRA 40, Zeiss) with 20 kV electron beam energy. Evaluation of bone mineralization density distribution (BMDD) was performed on images with 1.8 μm/pixel lateral resolution and osteocyte lacunae sections (OLS) analysis on qBEI images obtained with 0.9 μm/pixel lateral resolution (Mahr et al., 2021).

### 2.2 Fibroblast cell culture

For the isolation and culture of patient fibroblast cells, a skin biopsy was taken from the patient, immediately washed with 70% EtOH and 1× PBS, cut into small pieces and placed in a culture dish with cutting edges facing the bottom, containing 1× DMEM complete medium (10% FCS + 1% Glutamax +1% antibiotic-antimycotic), under sterile conditions. Fibroblasts grew out of the tissue within several weeks. Culture medium was changed once in a week and cells were split at 80% confluency. Control fibroblast were isolated from healthy individuals, that match the age and gender of the patient.

### 2.3 Whole exome sequencing

WES was performed from peripheral-blood DNA samples as described previously (Kremer et al., 2016). Briefly, using a SureSelect Human All Exon V6 kit (Agilent) coding regions were enriched and followed by sequencing as 100 base-pairs, paired-end on an Illumina NovaSeq 5000. Using human reference genome (UCSC Genome Browser build hg 19) reads were aligned using Burrows-Wheeler Aligner (v.0.7.5a) (Li and Durbin, 2009). SAM_tools (version 0.1.19) were used to detect single nucleotide variants and small insertions and deletions (Genome Project Data Processing, S, et al., 2009).

### 2.4 Targeted sanger sequencing

A 498 base-pair (bp) PCR product corresponding to exon 50 and adjacent intron regions of *COL1A2* (NM_000089.4) was amplified using the primer pair COL1A2-Ex50-F 5′-AGGGGAGGGAAGGAACTGT-3′ and COL1A2-Ex50-R 5′-GACAAAATTGGAACCCAGGA-3′ and GeneAmp™ Fast PCR (ThermoFisher) with 1 min at 95 °C followed by 40 cycles of 96 °C for 15 s, 60 °C for 30 s, and 72 °C for 40 s. The purity and length of the PCR product was checked by agarose gel electrophoresis and a 2 μl aliquot was treated by ExoSAP-IT (ThermoFisher) followed by BigDye™ Terminator v3.1 Cycle Sequencing (ThermoFisher).

### 2.5 qRT-PCR

RNA was isolated from patient fibroblasts in 180 ccm culture flasks grown to 2/3 confluency using TRI Reagent™ (ThermoFisher). Reverse transcription was performed using Maxima Reverse Transcriptase and oligo-dT priming. Quantitative real-time was performed with SYBR® Green Master Mix (Bio-Rad) and the following forward primers: (1) COL1A2-E49-F 5′-CTCGCTCAGCACCTTCTCTC-3′ recognizing wild-type and splice mutation, (2) COL1A2-E49 + 50-F 5′-CCAGAGTGGAGCAGTGGTTA-3′ recognizing the wild-type splice site, and (3) COL1A2-E49 + 50a-F 5′-GAGCAGTGGCGAAACCTGTA-3′ recognizing the mutated splice-site. The reverse primer was COL1A2-E51-R 5′-ATGCAATGCTGTTCTTGCAG-3′ for all three reactions. Cycling conditions for qPCR were 3 min at 95 °C followed by 40 cycles of 98 °C for 2 s, 63 °C for 40 s, and 72 °C, 60 s. Melting curves were determined after that.

### 2.6 Protein preparation and western blot analysis

Protein preparation and western blots were carried out using standard procedures. Briefly, cell lysates were prepared after washing the cells thrice with cold 1xPBS and lysed in RIPA buffer (Cell Signaling) supplemented with 100× protease inhibitor cocktail (Sigma-Aldrich) and 100× phosphatase inhibitor cocktail (Sigma-Aldrich) and 200× PMSF (Cell Signaling). Proteins were extracted from cell lysates by centrifugation at 14000 ×*g* for 10 min at 4 °*C. medium* samples were supplemented with 25× protease inhibitor cocktail (Sigma-Aldrich). Proteins were concentrated in an Ultra-15,100 K filter device by centrifuging at 5000 ×*g* for 30 min. Protein concentrations were determined via the BCA protein assay (Thermo Scientific, Rockford, IL, USA). Afterwards, equal amounts of proteins (~20 μg) were loaded onto 4–20% gradient gels (Bio-Rad), separated by gel electrophoresis, and electrotransferred onto PVDF membranes (Bio-Rad). The membranes were then incubated with blocking reagent (Bio-Rad) for 10 min. Membranes were then incubated with the following primary antibodies diluted in blocking buffer (Bio-Rad) overnight: monoclonal anti-rabbit COL1A1 antibody (dilution 1:1000; Cell Signaling), polyclonal anti-rabbit COL1A2 antibody (dilution 1:1000, Abcam), rabbit monoclonal actin antibody (1:2000, Cell Signaling). Membranes were then washed thrice with 1xTBS 0.1% Tween. Detection was carried out using the corresponding peroxidase-conjugated (HRP) secondary antibodies (dilution 1:15000, Cell Signaling) diluted in blocking buffer (Bio-Rad), with incubation for 1 h at room temperature (RT). The membranes were washed six times with 1xTBS 0.1% Tween and developed using enhanced chemoluminescence (ECL, Cell Signaling). For functional studies, patient and control fibroblast cells were treated with ascorbic acid 0.05 μg/ml for 20 h and then medium was harvested, cells were lysed and COL1A2 was detected by western blotting.

### 2.7 Statistical analysis

To detect statistically significant differences between patient and controls, two-sided unpaired Student's *t*-tests. P-values <0.05 was considered for statistically significant differences. For statistical evaluation, the GraphPad Prism 8 was used.

## 3 Results

### 3.1 Clinical report

**Fig. 1:**
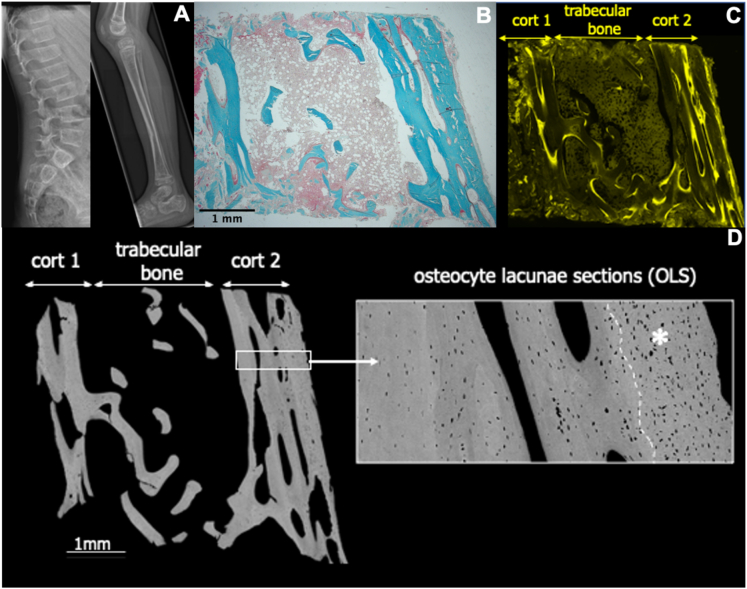
Bone imaging and biopsy data. *A*) X-rays show L1 vertebral fracture and left tibia fracture. *B*, *C*, *D*) Overview on the bone biopsy sample. *B)* Histological section, Goldner stained (green: mineralized matrix, red osteoid), *C)* Fluorescence image showing fluorescence labels showing sites with new bone formation *D*) quantitative Backscattered electron microscopy image (BEI) of the transiliac bone biopsy sample cross-section. *B* and *D*) demonstrate reduced trabecular number (with increased thickness) but normal cortical thickness *D*) qBEI images showing hyperosteocytosis (insert: detail of the periosteal site of corticalis 2) Cort 1: corticalis 1, Cort 2: corticalis 2, broken line delineates area with primary bone (white asterisk).

A five-year-old boy presented to our tertiary hospital with a history of recurrent low impact extremity long bone fractures from age 2.5 years and motor delay from age 9 months for which he had received orthotic lower extremity casts. He was born to non-consanguineous Caucasian parents. On clinical examination he demonstrated joint hyperlaxity mainly affecting the upper extremity, grey sclera, mild facial dysmorphism and ginger hair but no leg bowing or dentinogenesis imperfecta. At age 5, his growth was impaired with a height z-score of −2.25 and dual energy X-ray absorptiometry scanning revealed reduced bone mineral density at the lumbar spine (z-score −4.6) and total body (z-score −4.3). Lateral spine imaging revealed an L1 vertebral fracture (Fig. 1). The patient was diagnosed with OI, had a diagnostic bone biopsy and was started on intravenous bisphosphonate therapy to which he responded well. To confirm the clinical diagnosis of OI, a gene panel analysis was conducted but no abnormalities were found. This was followed by WES in a centre of expertise, but no causative or suspicious gene variant could be detected, which lead us to repeat WES and conduct further research.

### 3.2 Bone biopsy results

Bone histomorphometry was largely typical for OI type I as it showed rather low trabecular number (1.4 per millimeter of cross-section versus 1.77 ± 0.31 in age-matched controls (Glorieux et al., 2000)), four-time increased cortical porosity (27.8% versus about 7% in heathy children (Rauch et al., 2007))*,* low mineral apposition rate (0.74 μm/day) and low adjusted apposition rate (0.18 μm/day) reflecting a diminished matrix production by osteoblasts per unit of time (Rauch et al., 2000)*.* However, the mean cortical width (0.69 mm) was within normal range and trabecular thickness (133.5 μm) was not reduced on the sample. The few rather isolated trabeculae were quite thick and thus the overall value for trabecular bone volume was numerically within normal range, although microarchitecture was profoundly disturbed (Fig. 1) (Glorieux et al., 2000). qBEI analysis did not reveal elevated density of mineralization (average mineral density, CaMean in trabecular bone: 22.16 wt% calcium versus 22.48 ± 0.73 in controls; in cortical bone, 20.97 wt% calcium versus 21.86 ± 1.15 in controls (Mahr et al., 2021)). The density of the osteocyte lacunae (OLS) was substantially elevated which is typical for OI type I (trabecular bone: +17%; cortical bone: +212% versus control values including primary bone or + 27% excluding primary bone). Due to larger OLS-area (trabecular bone: +47%; cortical bone: +70%), OLS porosity was increased even beyond values established for OI type I bone (trabecular bone: 0.91% versus 0.76%; cortical bone: 1.39% or 2.27% respectively, without or with primary bone versus 0.88% in OI type I (Mahr et al., 2021))*.*

### 3.3 Identification of a cryptic splice site mutation *in COL1A2*

**Fig. 2:**
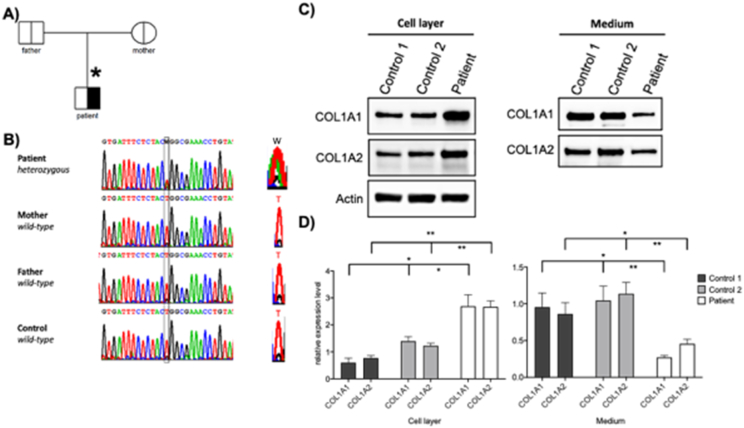
Validation of the *COL1A2* mutation by Sanger sequencing of genomic DNA and expression studies of COL1A1 and COL1A2. *A)* Family pedigree with wt *COL1A2* in parents and heterozygous mutation in the patient. Asterisk indicates individual studied. *B)* Sanger sequencing of the gDNA of the patient, parents and healthy control shows that the de novo mutation is not detectable in the parents. W = A and T. On the right side, magnification of the chromatograms of the position c.3597 is shown. *C)* Western blot analysis of COL1A1 and COL1A2 expression in patient and two different control fibroblasts in whole cell lysates and extracellular media after treatment with ascorbic acid 0.05 μg/ml. *D)* quantification analysis of western blot using Bio-Rad Image Lab 6.1 software. Columns represent the mean of at least three independent experiments; *bars*, *standard* error of the mean (SEM); *significant (p < 0.05), or **highly significant (p < 0.005) differences obtained by comparing each of the control group and the patient.

Since the clinical exome gene panel with all known OI genes as well as WES at a centre of expertise had revealed no pathogenic variant, WES was performed again and deemed no pathogenic variant. However, manual screening of *COL1A1* and *COL1A2* was conducted, which finally revealed the cryptic splice site *COL1A2* variant c.3597 T > A. This novel *COL1A2* variant was shown to be absent in blood samples from the parents by Sanger sequencing (Fig. 2A). The de novo heterozygous mutation c.3597 T > A substitution is located in the 50th exon in the C-terminal propeptide of *COL1A2* (Supplementary Fig. 1 and Supplementary Table 1) and does not change the amino acid sequence but creates a new splice site.

There are several lines of evidence supporting that this mutation creates a new acceptor splice site. This includes (i) the variant is de novo and absent in control, (ii) the variant is predicted computationally to create a new splice site using three splice site prediction programs: (https://www.fruitfly.org/seq_tools/splice.html, http://wangcomputing.com/assp/, http://www.cbs.dtu.dk/services/NetGene2/), and (iii) the typical clinical, imaging and bone histomorphometric phenotype of OI in this patient (Fig. 1).

The new splice acceptor results in a deletion of 24 amino acids from p.1177 to p.1200 in the C-terminal part of the *COL1A2* C-propeptide (Supplementary Table1). To elucidate the underlying cellular pathogenesis, we analyzed the expression and secretion of COL1A2 protein in the patient fibroblasts and in the extracellular medium, compared to controls. Functional studies in the patient demonstrate cellular accumulation of COL1A1 and COL1A2 with reduced secretion to the extracellular medium (Fig. 2C) compared to controls. These data indicate that export of type I collagen is compromised in the fibroblasts of the patient.

### 3.4 Effect of *COL1A2* (NM_000089.4) c.3597T>A mutation on splicing

**Fig. 3:**
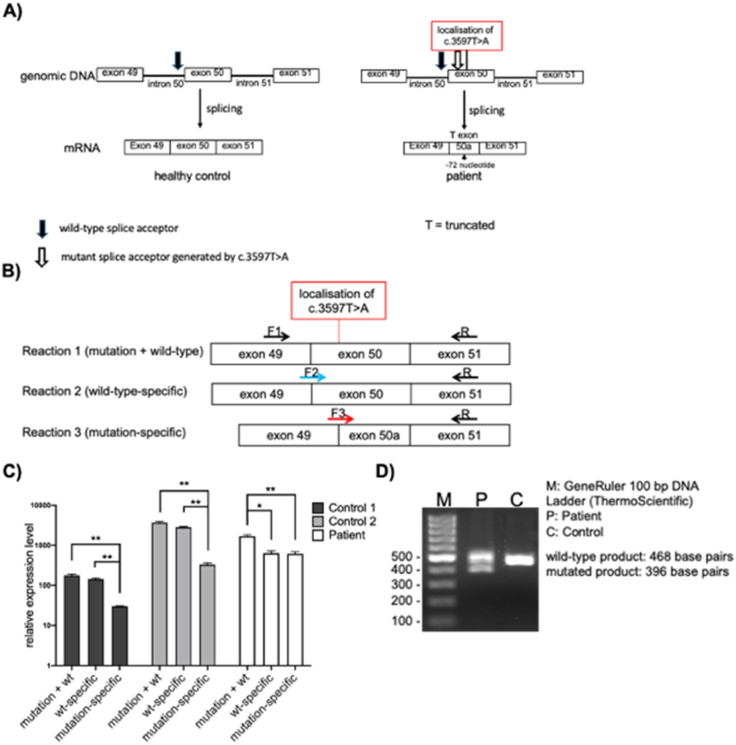
Effect of *COL1A2* (NM_000089.4) c.3597T>A mutation on splicing. *A)* Schematic representation of the investigated regions of the normal and mutant *COL1A2* transcripts. The mutation in exon 50 creates a new splice acceptor site, which results in the deletion of 72 nucleotides (24 amino acids). The black arrow marks the normal splice site, the white arrow the new acceptor splice site in the patient. *B)* Schematic representation of the three different forward primers amplifying mutation and wt (F1), only wt, bridging exon junction 49/50 (F2), only mutated exon boundary, bridging exon junction 49/50a (F3). Primers for cDNA amplification were designed using primer 3 algorithm (PRIMER3.UT.EE). Black arrows indicate primers amplifying both wt and mutant starting from the middle of the adjacent exons (49 and 51 exons). *C)* Quantification of *COL1A2* splice products in cDNA of the heterozygous patient compared to two healthy controls. The relative expression value shown in the figure is based on two housekeeping genes *HPRT1* and *RPL27*. In two healthy controls, the amounts of the reaction 1 (mutation + wt) and reaction 2 (wt only) are similar. In the patient, the wt specific reaction (reaction 2) is approximately 40% behind compared to wt and mutant (reaction 1). The equal amounts of PCR products of mutant- and wt specific (reactions 2 and 3, respectively) in the patient also confirms that the mutant mRNA is stable. Remarkably, a small amount of the mutant PCR product (5% of wt) can be amplified also in controls. *D)* Agarose gel wt product is 468 bp and mutated product is 396 bp and can be detected in the patient but not in the control. Primer used for this PCR is reaction 1.

To elucidate the effect of *COL1A2* cryptic splice site mutation c.3597 T > A on splicing, we designed appropriate forward primers amplifying either mutation and wild-type cDNA, or wild-type (wt) only, or mutated exon boundary only (Fig. 3A and B).

The qRT-PCR data showed almost no difference between mutation + wt and wt-specific primers in healthy controls but a statistically significant difference in patient cells (Fig. 3C). In patient cells, the amount of the wt-specific cDNA decreased to approx. 40%. Furthermore, the mutant specific PCR product is found in equal amounts as wt specific, indicating that wt and mutant mRNAs are present in equal amounts in the patient (Fig. 3C and D). This also confirms that the mutant mRNA is stable. Remarkably, a small amount of the mutant product (5% of wt) can be amplified also in the controls. Therefore, it seems that a cryptic splice acceptor already exists in the wt sequence of *COL1A2*. Through the mutation in the affected individual, this splice acceptor is highly enhanced and becomes the preferred splice acceptor site.

### 3.5 Consequences of the new *COL1A2* acceptor splice site on the reading frame and the amino acid level

**Fig. 4:**
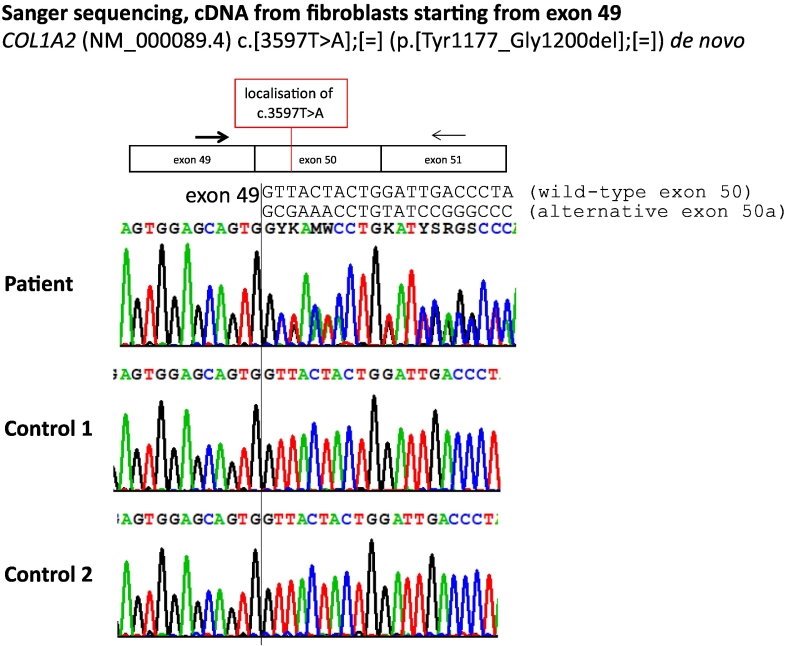
Consequences of the new *COL1A2* acceptor splice site on the reading frame and the amino acid level. Sequencing of the cDNA from fibroblasts in the patient and two controls. The cDNA was amplified by the primers shown in Fig. 3. Reaction 1 amplifying wt and mutant alleles. The vertical black line in the diagram shows the end of the exon 49. In the patient's sequence mentioned above Y: C or T; K: G or T; M: A or C; W: A or T; S: G or C; R: A or G.

To analyze the effect of *COL1A2* acceptor splice site mutation on the reading frame and the amino acid level, we sequenced the cDNA of patient and control fibroblasts using mutation and wt specific primers (Fig. 3B). Sanger sequencing of the cDNAs revealed that one nucleotide change created in the case of the patient, at the beginning of exon 50a, two sequences in parallel and it seems as if both sequences are present in the same amount (Fig. 4). Both controls show the wt sequences exclusively (Fig. 4). The small amount of the cryptic splice product in the healthy control found in qRT-PCR (Fig. 3C) is not detectable here. The new splicing in the patient results in a truncation of exon 50, excluding the first 72 nucleotides (24 amino acids). This in-frame deletion results in a stable mRNA (Supplementary Fig. 2). The new COL1A2 protein is lacking the amino acids from tyrosine 1177 to glycine 1200.

Sanger sequencing of cDNA starting from the exon 49–50 junction, using wt specific primers reaction 2 (Fig. 3B), showed that only the T can be seen at the mutant position site c.3597 in the patient (Supplementary Fig. 3). This indicates that the wt splice acceptor site is not used to a substantial amount. Otherwise, T and A would be seen in equal amounts as in the genomic DNA of the patient (Supplementary Fig. 3). On the mutant allele exon 50 contains both the wt splice site and the adjacent new acceptor site generated by the mutation. Obviously, the spliceosome predominantly decides to take the new acceptor site. This indicates that the new acceptor site is much more attractive than the wt acceptor site. The comparison with gDNA shows the amount of mutation where T and A present roughly in equal amounts in the patient.

## 4 Discussion

Here we confirm the pathogenicity of the novel autosomal dominant *COL1A2* mutation c.3597T>A (NM_000089.4) as a new, cryptic splice site mutation, representing a type III splicing defect (Abramowicz and Gos, 2018). The clinical and bone histomorphometric features of the affected patient with increased cortical porosity, disturbed trabecular architecture, hyperosteocytosis were compatible with a mild to moderate form of OI phenotype, although bone matrix mineralization was not increased. Moreover, the observed reduction in bone mineral apposition rate and adjusted apposition rate indicate that osteoblasts produce an abnormally small amount of matrix per unit time, in line with the performed Western blot analysis showing a marked reduction in collagen secretion by fibroblasts. We further demonstrate that this highly unusual mutation does not change the affected amino acid sequence but activates a cryptic splice donor site that leads to abnormal splicing, an in-frame deletion of 24 amino acids in the COL1A2 protein. This splice acceptor is highly enhanced and becomes the preferred splice acceptor site. Notably, *COL1A2* has 1366 amino acids, and the 24 amino acid in-frame deletion in the affected individual only accounts for a 1.7% difference in size. Hence, there is limited difference in COL1A2 size at protein level by western blotting.

To our knowledge this is the first cryptic splice site mutation observed in a coding exon of the collagen type 1 coding genes (*COL1A1*, *COL1A2*). Thereby, this opens a new category of mutations to be screened for in much more detail as standard analyses are usually unable to detect these cryptic splice site mutations. Functional analysis revealed that the COL1A2 protein is accumulating intracellularly and its transport to the extracellular medium is reduced, suggesting that the deletion of the 24 amino acids in the C-propeptide are required for the transport of COL1A2 to the extracellular space. Our results suggest that the collagen fibrils are of poor quality. Notably, the C-propeptide makes up amino acid position 1120–1366 and the deletion found is the patient is from 1177 to 1200, hence about 10% of the C-propeptide is lost.

To date, modern sequencing technologies allow more than 95% of OI cases to be genetically resolved. Here we demonstrate that a negative genetic screening result does not necessarily indicate the involvement of unknown disease genes. Special attention should be paid to any kind of de novo variants even if they have low scoring or they do not change the amino acid sequence. In order to recognize de novo variants trio sequencing is highly recommended. The search for the genetic cause can take variable approaches. The first approach should include OI gene panels. If a gene panel does not reveal a pathogenic variant, an exome wide analysis using WES or WGS, analyzing all genes in human genome, should be considered. If you apply trio sequencing, WES offers, in most cases, the possibility to identify a de novo variant or variants in unknown genes. However, false negative interpretations of WES screening might potentially arise from inadequate analysis of the data. Also, the result of WES screening might look negative at first glance and only after careful, detailed analysis of the known OI genes, cryptic mutations e.g. those introducing a new acceptor splice site as in this case, can be discovered both the intronic as well as the coding regions. Trio sequencing, so parents are also sequenced, should be also considered. Conducting this kind of detailed analysis of known OI genes (El-Gazzar and Hogler, 2021) (https://databases.lovd.nl/shared/genes/COL1A2) may well further increase the overall detection rate in subjects with OI. So far, there are 730 pathogenic variants that have been identified in *COL1A2* (https://databases.lovd.nl/shared/variants/COL1A2/unique?search_var_status=%3D%22Marked%22%7C%3D%22Public%22). Among these, ~11% variants were reported as alterations in splice-sites representing the second most common type of mutation. To the best of our knowledge, no type III splicing mutation has been described.

Splicing defects are rare but well recognized types of mutation but there are various types. Cryptic splice site mutations as in this case, so-called type III splicing mutations (Abramowicz and Gos, 2018) appear to be rare but the true frequency is not well known as they may easily escape detection. Other recent examples for similar cryptic splice site mutations have been published in a number of rare diseases (Daidone et al., 2020; de Boer et al., 2019; Habara et al., 2009; Wang et al., 2018). They all have in common that they were not easily detected, hence they may be more common than they are known. Therefore prediction algorithms for splicing defects (Abramowicz and Gos, 2018) require further optimization (Habara et al., 2009).

## 5 Conclusion

This thoroughly investigated case highlights a new category of mutations found in *COL1A2*. We describe here a cryptic splice site mutation in exon 50 of *COL1A2* that results in decreased secretion of COL1A2 and causes OI. The fact that the mutation was only found by manual screening of WES data highlights the limitations of the analytical pipelines to identify such cryptic splice sites and highlights the importance of trio-sequencing in OI.

## Funding

JAM is supported by the 10.13039/501100002428Austrian Science Fund (FWF) [I 4695-B, GENOMIT]. This work was also supported by the Austrian Social Health Insurance Fund (OEGK) and the 10.13039/501100007146Austrian Workers' Compensation Board (AUVA) and the German Federal Ministry of Education and Research (BMBF) grant to the German Network for Mitochondrial Disorders (mitoNET, 01GM1906D) and by BMBF and Horizon2020 through the E-Rare project GENOMIT (01GM1920A). All authors are employed by the indicated institutions.

## CRediT authorship contribution statement

AEG designed the current study and oversaw the laboratory analysis, interpreted lab data and drafted the manuscript. HP and JAM conducted and analyzed the WES that identified the mutation. KB performed the qRT-PCR and the sanger sequencing. BV performed the western blotting. KT summarised the clinical data. HCD organized all genetic testing. NFZ and SB conducted and interpreted bone biopsy experiments. WH made the diagnosis, arranged all clinical and research laboratory testing, performed the bone biopsy, supervised the writing of the manuscript and gave final approval for submission. All authors provided intellectual input and approved the submitted version.

## Declaration of competing interest

None.

## Appendix A Supplementary data

Supplementary material Image 1
