# Supplementary material for: A novel cryptic splice site mutation in COL1A2 as a cause of osteogenesis imperfecta
Source: Bone Rep. 2021 Jul 26;15:101110. doi: 10.1016/j.bonr.2021.101110 (PMC8339231; doi:10.1016/j.bonr.2021.101110)
Supplement: Supplementary file 1 — Supplementary material [file mmc1.docx]

**9. Supplementary table**

**9.1. Supplementary Table 1**: Detailed information of variant in *COL1A2*

| **Position** | **Gene** | **Mutation** | **OMIM** | **Type** | **Population MAF** |
| --- | --- | --- | --- | --- | --- |
| (hg19) chr7:94,057,675 | *COL1A2 (*NM_000089.4) | 4 c.[3597T>A];[=] (p.[Tyr1177_Gly1200del];[=]) | 120160 | Silent, altering splicing | 0 |

**9.2. Supplementary figures**


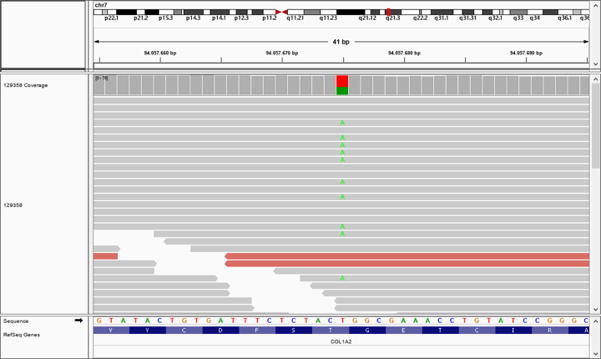


**Supplementary Figure 1.** ***COL1A2* variant identified by whole-exome sequencing.** Whole exome sequencing of a 5-year-old male patient with OI. Screenshot from the integrative genomic visualization (IGV) program showing the WES reads mapped to reference genome at the COL1A2 locus. The mutation T at position 3597 changed to A, indicated in green colours in the diagram. The mutation was found in 41% of the reads, the coverage was 70-fold.

>wild-type

agtgaaaatgcatttgggtaaagattacattatgtgaaatcacacccaattaatggagcgtcatcttctcccaaccagcacccaacctcatttcccttaaaatgtatttttgcacttttcatagtaataagtaccctgatttgatttttcatggaggaggggagggaaggaactgtctaatcttaaaaatagccaccctcttcctcttaaatatggggtagacaatcaaaaatgttacttatgagagtcagtatctttcattagttattattagaatctgtgttctgctcaatgagaagtttcatgatctgaatgttattttcttaaaagGTTACTACTGGATTGACCCTAACCAAGGATGCACTATGGATGCTATCAAAGTATACTGTGATTTCTCTACTGGCGAAACCTGTATCCGGGCCCAACCTGAAAACATCCCAGCCAAGAACTGGTATAGGAGCTCCAAGGACAAGAAACACGTCTGGCTAGGAGAAACTATCAATGCTGGCAGCCAGgtgaggaatcccacaaacacctctccttctgctaaataatattttggtaggactgtttgttaattatctgcattttaatctctgacaaaaatgggcttattaaaaaaagacctgttcctttcctgggttccaattttgtcctaaattgcacattagaagatggattgattggacacatccatgtaattcaaagttattattcaaatttgacttaattggtaatcattgaaaaaactgactaatgtcatttagtgtgaaggagcactggccagctatatgccacactcatacatatgcattttcagaatgtgagcagcttttctgaatttttaatcaaaccttttcaccaactttactgaatgcctactggaattccataaattacaaaatgacagaaaaaga

>c.3597T>A

agtgaaaatgcatttgggtaaagattacattatgtgaaatcacacccaattaatggagcgtcatcttctcccaaccagcacccaacctcatttcccttaaaatgtatttttgcacttttcatagtaataagtaccctgatttgatttttcatggaggaggggagggaaggaactgtctaatcttaaaaatagccaccctcttcctcttaaatatggggtagacaatcaaaaatgttacttatgagagtcagtatctttcattagttattattagaatctgtgttctgctcaatgagaagtttcatgatctgaatgttattttcttaaaagGTTACTACTGGATTGACCCTAACCAAGGATGCACTATGGATGCTATCAAAGTATACTGTGATTTCTCTACaGGCGAAACCTGTATCCGGGCCCAACCTGAAAACATCCCAGCCAAGAACTGGTATAGGAGCTCCAAGGACAAGAAACACGTCTGGCTAGGAGAAACTATCAATGCTGGCAGCCAGgtgaggaatcccacaaacacctctccttctgctaaataatattttggtaggactgtttgttaattatctgcattttaatctctgacaaaaatgggcttattaaaaaaagacctgttcctttcctgggttccaattttgtcctaaattgcacattagaagatggattgattggacacatccatgtaattcaaagttattattcaaatttgacttaattggtaatcattgaaaaaactgactaatgtcatttagtgtgaaggagcactggccagctatatgccacactcatacatatgcattttcagaatgtgagcagcttttctgaatttttaatcaaaccttttcaccaactttactgaatgcctactggaattccataaattacaaaatgacagaaaaaga

**Supplementary Figure 2. Sequence of Exon 50 in wt and mutant.** Lower case letters are intron sequences. Upper case are exon 50 sequences. An "AG" before the beginning of the exon sequence is the canonical part of most splice acceptor sites. The underlined sequence highlights the deletion caused by the new splice acceptor site. The new splice acceptor site leads to a 72 nucleotide (24 amino acid) in-frame deletion.

**Supplementary Figure 3.** **Sanger sequencing of cDNA from control and patient fibroblast cells starting from the exon 49-50 junction.** mRNA of the patient and the two controls were isolated and cDNAs were generated. Sequencing of reaction 2 (see Fig. 3*B*, wt specific) and at the position c.3597 only shows that the T can be seen in the patient cDNA. This means that the wt splice acceptor site is not used in the mutant allele in significant amounts. The comparison with gDNA shows the amount of mutation where T and A are present roughly in equal amounts.
